# Explainable machine learning-based preliminary screening for viral encephalitis by blood routine analysis

**DOI:** 10.3389/fneur.2026.1844506

**Published:** 2026-06-19

**Authors:** Bo Lv, Jie Pan, Aiming Shi, Dongxing Wang

**Affiliations:** 1 Department of Pharmacy, The Second Affiliated Hospital of Soochow University, Suzhou, China; 2 ‌Department of Neurology‌, The Second Affiliated Hospital of Soochow University, Suzhou, China

**Keywords:** machine learning, prediction, routine blood analysis, viral encephalitis, XGBoost

## Abstract

**Background:**

Viral encephalitis (VE) is a severe neurological emergency; however, timely diagnosis remains challenging, particularly in resource-limited settings. This study aimed to develop and validate an interpretable machine learning (ML) model for the preliminary risk stratification of VE based solely on routine blood analysis (RBA).

**Methods:**

A retrospective cohort of patients (*n* = 313, train/test = 8/2) with suspected VE was collected from the electronic health records of a tertiary hospital. ML models, including K-nearest neighbor (KNN), light-gradient boosting model (GBM), logistic regression, random forest, supporting vector machine (SVM), and extreme gradient boosting (XGBoost), were trained and validated using demographic and hematological features. The Shapley Additive Explanations (SHAP) framework was used to ensure model transparency and interpretability.

**Results:**

The XGBoost model demonstrated superior performance with an AUC of 0.949 (95%CI: 0.921 ~ 0.978) in 10-fold cross-validation in the train set and 0.900 (95% CI: 0.801–1.000) in the test set. SHAP analysis identified serum albumin (ALB) and white blood cell (WBC) counts, and low neutrophil (NEU) counts were the most significant contributors to VE prediction. Notably, the interactions between ALB and WBC were also highly influential in VE prediction.

**Conclusion:**

This study presents an accurate and explainable XGBoost model for VE preliminary screening. Based on universally available blood indicators, it serves as a practical front-end tool for optimizing diagnostic workflows in emergency or primary care settings.

## Introduction

Encephalitis, an inflammatory condition of the brain parenchyma, represents a significant neurological emergency characterized by fever, altered mental status, seizures, and focal neurological deficits (1). Viral encephalitis (VE) constitutes the predominant form of infectious encephalitis, accounting for approximately 70% of confirmed cases, with a reported global incidence of 1.4 to 13.8 per million person-years (2). The 2025 WHO guidelines underscore the critical importance of rapid diagnosis for effective management and improved patient outcomes. However, achieving timely diagnosis remains a formidable challenge in clinical practice, particularly in primary and resource-limited healthcare settings.

Current diagnosis of VE primarily relies on clinical manifestations, electroencephalography (EEG), routine cerebrospinal fluid analysis (RCSFA), and neuroimaging (3). However, diagnosis based solely on clinical manifestations is limited by subjectivity (4). EEG, RCFA, and neuroimaging are ancillary examinations, which cost extra charges and are not rapid enough (5). Routine blood analysis (RBA) offers a universally available, cost-effective, and rapid alternative. Thus, to achieve‌ rapid diagnosis of VE, a preliminary risk screening tool based on clinical manifestations and RBA was needed, especially in primary medical institutions.

While the sensitivity and specificity of RBA are limited when used in isolation for diagnosing VE, integrating multiple indicators can improve diagnostic performance. Machine learning (ML) presents a powerful solution to this challenge (6). The ML approach enables a systematic and integrative assessment of disease risk by synthesizing complex, non-linear interactions within a comprehensive set of clinical variables (7–9). This approach moves beyond the fragmented evaluation of individual indicators, potentially unlocking the latent predictive power of RB. Despite its promise, the “black-box” nature of many ML models can impede their clinical adoption, as the lack of interpretability may undermine clinicians’ trust.

To address this gap, our study aimed to develop and validate an ML model for the preliminary risk stratification of patients with suspected VE based on admission RBA. Crucially, we used Shapley Additive Explanations (SHAP) values to construct an explanatory framework for our model. This dual approach not only seeks to provide a practical screening tool but also ensure transparency and foster clinical credibility by elucidating the contribution of each feature to individual predictions.

## Methods

### Data collection

Information on patients with suspected VE was collected from the electronic health records (EHRs) of the Second Affiliated Hospital of Soochow University between January 2010 and June 2025. Patients who underwent lumbar puncture (with an interval from symptom onset to lumbar puncture of ≤2 weeks) for symptoms such as dizziness, neuralgia, or insomnia were collected in our study, including one of the following (suspected as VE): (1) fever (axillary temperature ≥38 °C) within 72 h before or after presentation; (2) prodromal symptoms (e.g., nasal congestion, rhinorrhea, and myalgia) before the onset of neurological symptoms; or (3) electroencephalography findings suggestive of encephalitis.

The diagnosis of VE complies with the International Encephalitis Consortium diagnostic criteria. The primary criterion must be satisfied: alteration in consciousness or mental status (including decreased level of consciousness, lethargy, or personality change) persisting for ≥24 h, without another clear explanation. Secondary Criteria must include more than three of the following items: (1) fever (axillary temperature ≥38 °C) occurring within 72 h before or after presentation, (2) new-onset generalized or focal seizures, (3) new-onset focal neurological deficit signs, and (4) cerebrospinal fluid (CSF) white blood cell (WBC) count ≥5/mm^3^ [If significant red blood cell (RBC) contamination is present, use the correction formula: true CSF_WBC = measured CSF_WBC-(blood WBC × CSF_RBC)/blood RBC]. (5) Neuroimaging (MRI or CT) showing new or acute parenchymal abnormalities consistent with encephalitis. (6) Electroencephalogram (EEG) shows encephalitis-suggestive abnormalities not explainable by other causes.

Patients without cerebrospinal fluid testing were excluded due to the difficulty in establishing an accurate diagnosis. Additionally, patients with other types of encephalitis—including bacterial, fungal, tuberculous, syphilitic, parasitic infections, or autoimmune encephalitis—were excluded from this study.

Demographic characteristics and indicators of RBA were used to construct the ML models, including: sex, age, white blood cell (WBC) counts, lymphocyte (LYM) counts, neutrophil (NEU) counts, monocyte (MON) counts, albumin (ALB), and globulin (GLB) in serum. The results of EEG, RCSFA, and neuroimaging were used for diagnosis, but were not used to construct the ML models.

### Model training

The data were divided into a train set (80%) and a test set (20%) stratified by the response variable, whether the patient was suffering from VE. Ten-fold cross-validation and grid search were used to tune the hyper-parameters of the ML models. K-nearest neighbor (KNN), light-gradient boosting model (GBM), logistic regression, random forest, supporting vector machine (SVM), and extreme gradient boosting (XGBoost) were constructed as candidate models. The hyper-parameter of KNN was neighbors; the hyper-parameters of light-GBM were trees (number of trees) and n_min (minimum size of leaf node in tree-structure); the hyper-parameters of logistic regression were penalty and mixture; the hyper-parameters of random forest were trees and min_n; the hyper-parameter of SVM was rbf_sigma (Sigma of Radial Basis Function); the hyper-parameters of XGBoost were trees, learn_rate, and tree_depth.

**Figure 1:**
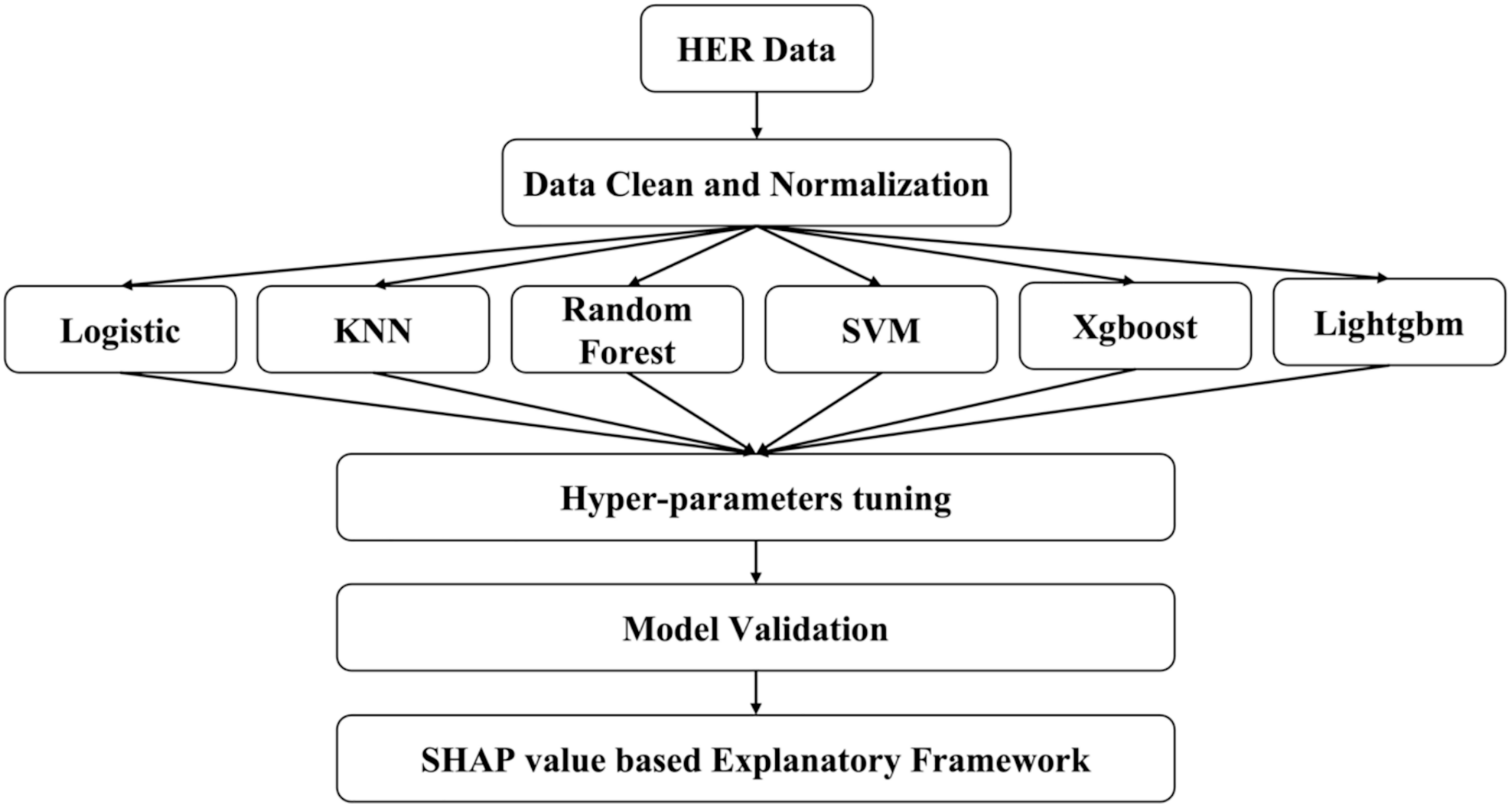
Workflow of model training and explaining.

Receiver operating characteristic (ROC) curve, calibrate curve, decision curve analysis (DCA) curve, and numerical indicators (including AUC of ROC, Brier, accuracy, sensitivity, specificity, and F1 score) were used to evaluate the performance of these models in both train set and test set, in which the threshold of classification was determined by the Youden index when calculating the sensitivity and specificity. The model comparison was performed using the DeLong test and net reclassification improvement (NRI). The calibrated metrics with a 95% confidence interval are shown in Supplementary Figures S1–S6. All of these models were trained using the tidymodels (version 1.0.0) framework in R software (version 4.2.2). The hyper-parameters were searched using the tune_grid() function in the tune package (version 1.3.0) with the default range. The explanatory framework for these ML models was constructed using SHAP values, including a bee swarm plot, a waterfall plot, a dependence plot, an interaction point plot, and an interaction heatmap. All of these SHAP values were calculated by the shapviz package (version 0.9.3) in R. The total workflow is shown in Figure 1.

### Statistical methods

The normally distributed numerical predictors were summarized as mean ± standard deviation, and the t-test was used to compare the difference between the patients with and without VE. The non-normally distributed numerical predictors were summarized as median (first and third quantile‌s), and a non-parametric test was used to compare the difference between the patients with and without VE. The categorical predictors were summarized as counts (percentages), and the chi-square test was used to compare the differences between patients with and without VE. The significance level was set at 0.05.

## Result

### Characteristics of patients

The baseline for these patients is shown in Table 1. It shows that the train set and the test set did not differsignificantly among these RBA indicators.

**Table 1:** Baseline of these patients.

Variables	Test (n = 63)	Train (n = 250)	*p*-value
Age	39 (30.5, 54.5)	35 (28, 52)	0.102
ALB, g/L	43.1 (40.8, 45.1)	42.6 (39.7, 44.8)	0.372
GLB, g/L	25.1 (22.15, 28.15)	25 (22.3, 27.6)	0.462
WBC counts, ×10^9^/L	5.2 (4.47, 6.1)	5 (4.4, 6.1)	0.480
LYM counts, ×10^9^/L	1.6 (1.2, 2.1)	1.7 (1.22, 2.2)	0.574
NEU counts, ×10^9^/L	4.6 (3.3, 5.9)	3.85 (2.9, 5.5)	0.094
MON counts, ×10^9^/L	0.5 (0.3, 0.7)	0.4 (0.3, 0.6)	0.101
VE, *n* (%)			1.000
No	16 (25%)	64 (26%)	
Yes	47 (75%)	186 (74%)	

### Model performance

**Figure 2:**
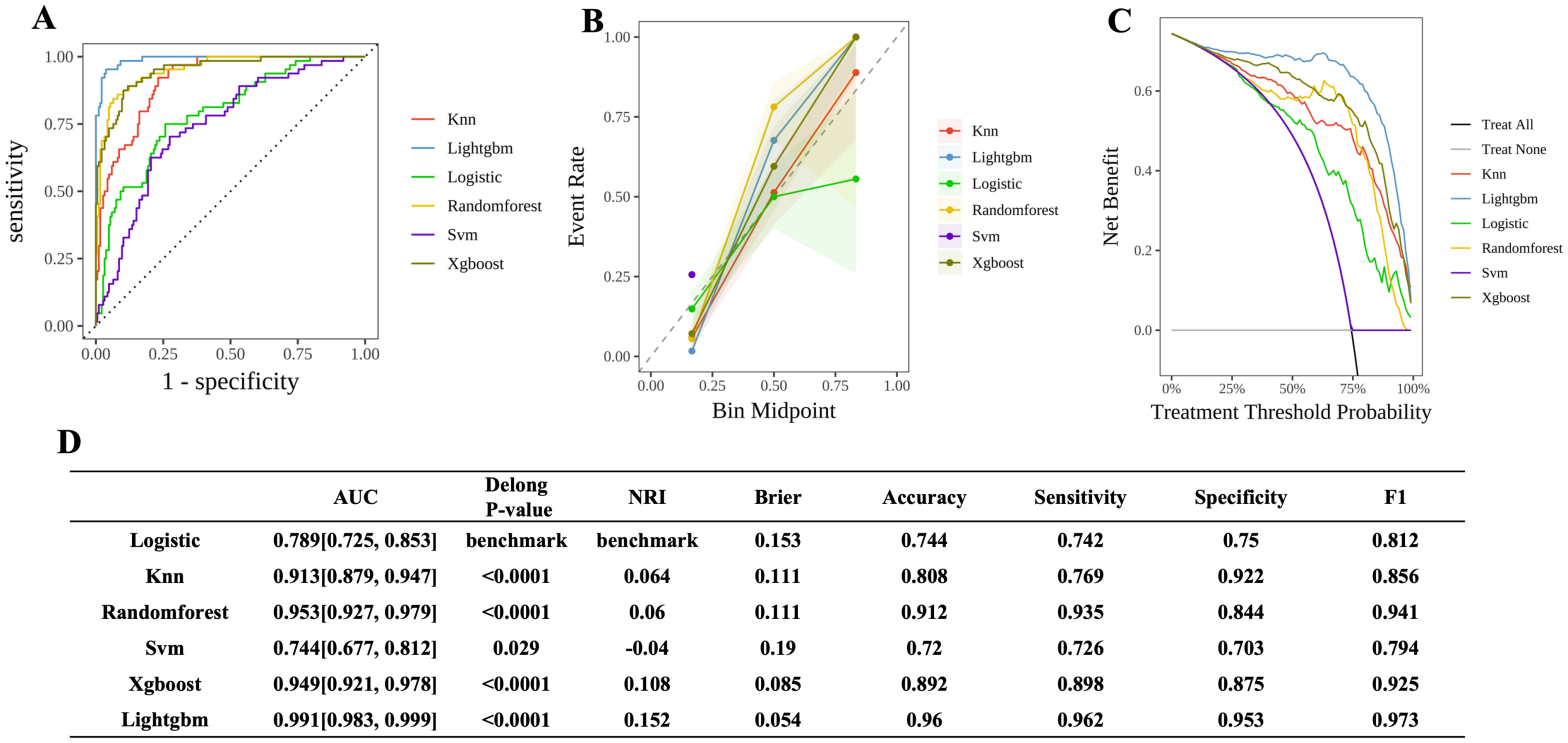
Performance of the ML models on the train set by 10-fold cross-validation: **(A)** ROC curve, **(B)** calibrate curve **(C)**, DCA curve **(D)**, and numerical indicators of model performance.

**Figure 3:**
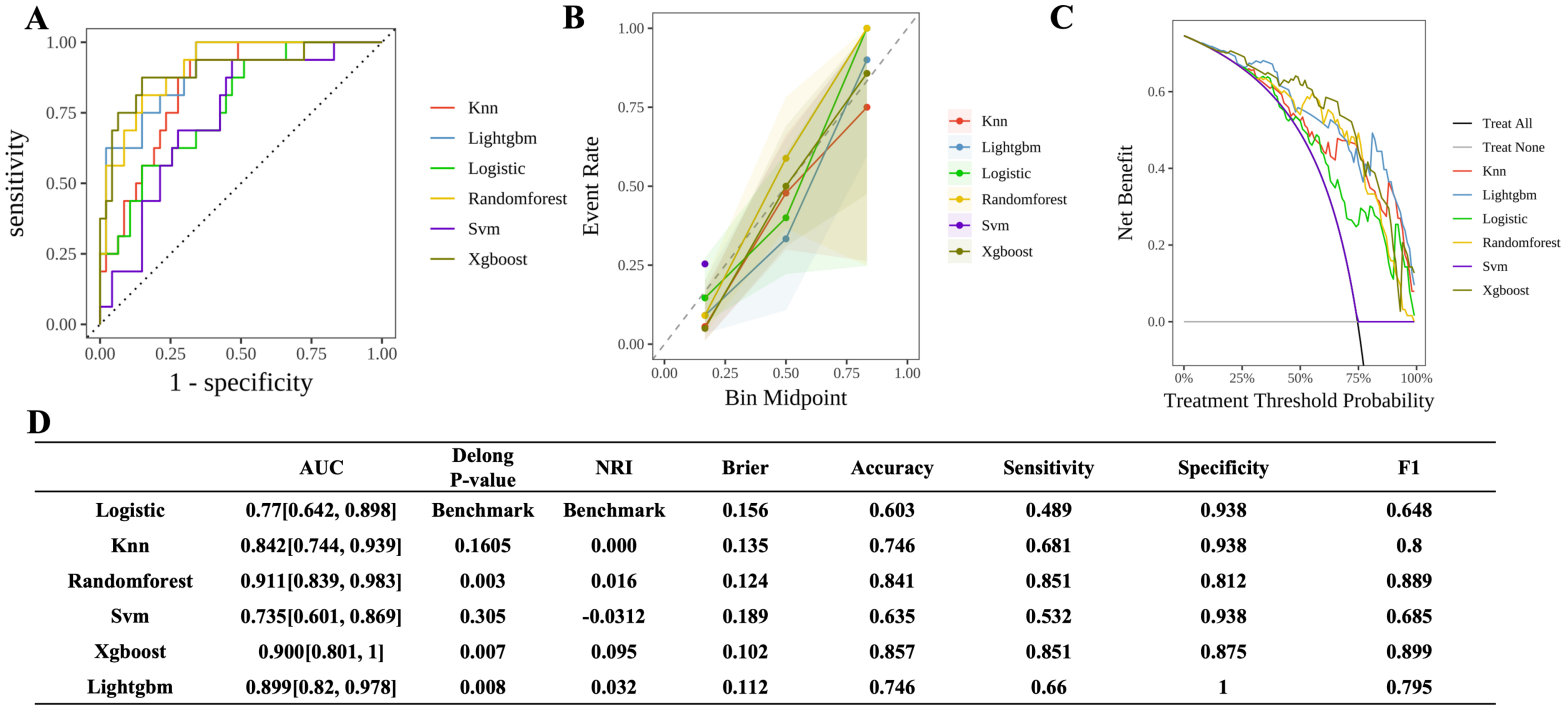
Performance of the ML models on the test set: **(A)** ROC curve, **(B)** calibrate curve **(C)**, DCA curve **(D)**, and numerical indicators of model performance.

In this study, KNN, light-GBM, logistic regression, random forest, SVM, and XGBoost were constructed as candidate models, of which the hyper-parameters were tuned by the grid-search method on 10-fold cross-validation. The performance of the best model for each type of ML approach is shown in Figure 2 (train set) and Figure 3 (test set).

According to the results, the performance of the random forest, light-GBM, and XGBoost models was excellent, with area under the curve (AUC) close to 0.9 on both the training and test sets. Furthermore, the calibration curve showed that the predicted probabilities of VE by the models were close to the actual probabilities of VE in both sets. The DCA curve also indicated positive net benefits across a wide range of threshold probabilities for VE diagnosis. The XGBoost model was selected as the final model due to the high AUC and the balance between sensitivity and specificity in the test set.

### Model explained

A SHAP-based explanatory framework was constructed to visualize how these predictors affect the final prediction. SHAP values represent the contribution of each predictor to the probability of VE. Consequently, a positive SHAP value represents the positive relationship between the predictor and VE probability, while a negative SHAP value indicates a negative relationship.

**Figure 4:**
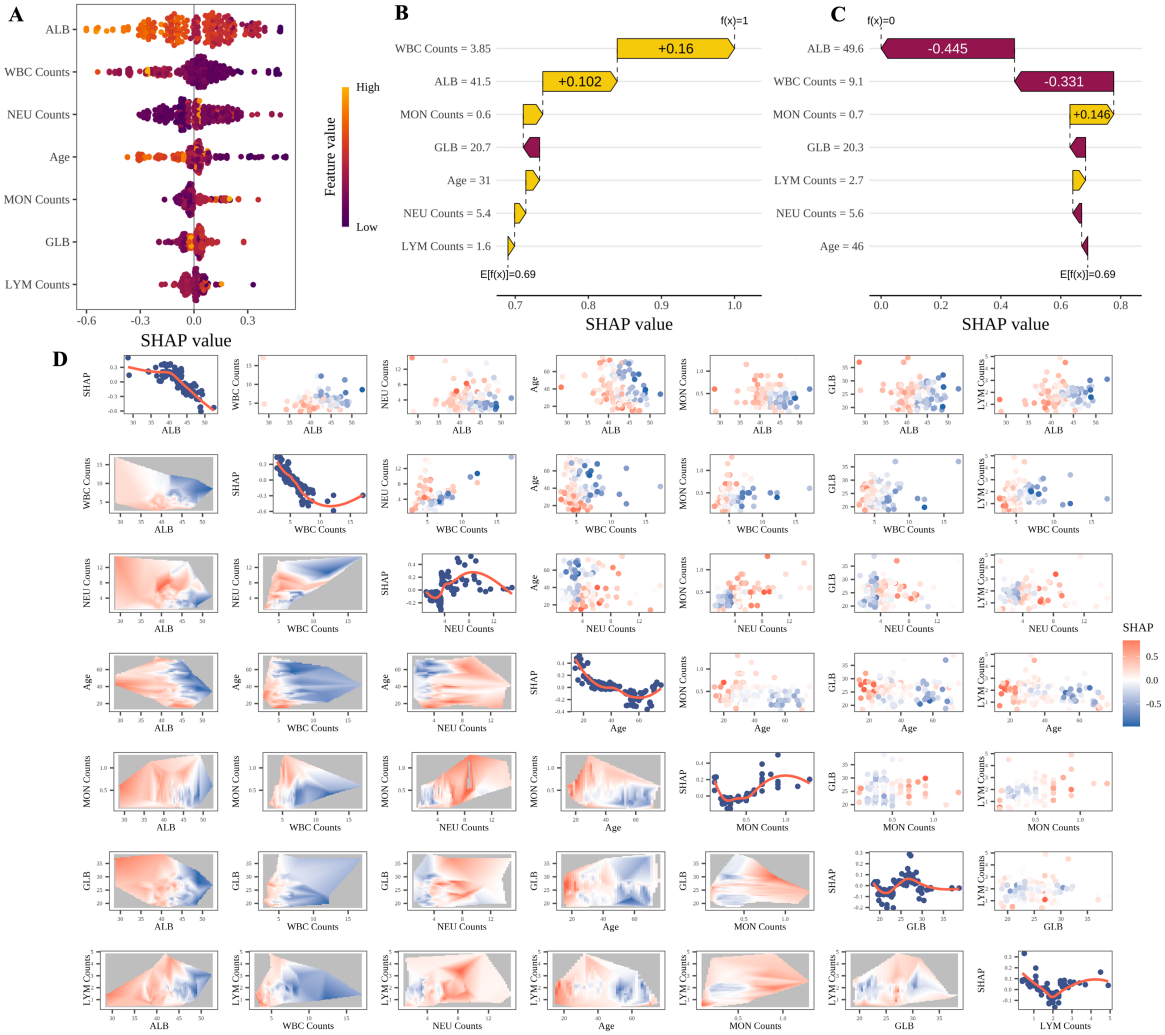
Explained frame for the XGBoost Model. **(A)** Bee swarm plot. **(B)** Waterfall plot for a typical VE patient. **(C)** Waterfall plot for a typical non-VE patient. **(D)** Interaction matrix. Diagnose: dependence plot; upper triangle: interaction scatter; lower triangle: interaction heatmap.

The overall contributions of these predictors were visualized by the bee swarm plot in Figure 4A. Waterfall plots were used to visualize how the model reached the prediction from values of predictors for a typical VE patient and a typical non-VE patient in Figures 4B,C, respectively. The interaction matrix, shown in Figure 4D, visualizes the marginal effect of each predictor and the interactions between each pair of these predictors. The explanatory framework was used to explain the XGBoost model.

As a result, it was found that the concentration of ALB and WBC counts in the blood was negatively related to the SHAP value. Conversely, NEU counts were positively related to the SHAP value when the level of NEU counts was low, suggesting that NEU counts contribute positively to the probability of VE in this range. Figure 4B represents a typical VE patient with low ALB (41.5 g/L, close to the 1st quantile) and low WBC counts (3.85 × 10^9^/L, lower than the first quantile). Figure 4C represents a typical non-VE patient with high ALB (49.5 g/L, higher than the third quantile) and high WBC counts (9.1 × 10^9^/L, higher than the third quantile).

## Discussion

In this study, the XGBoost model, which outperformed KNN, Light-GBM, Logistic regression, Random Forest, and SVM, was selected as the best model for predicting VE probabilities. Furthermore, a SHAP-based explanatory framework was constructed to improve the reliability of the XGBoost model. The AUC of the XGBoost model was 0.900 (95% CI = [0.801, 1.000]) on the test set, compared with 0.785 for an ordinary logistic regression model using the same RBA indicators (10). This demonstrated that ML approaches were better suited to handling the complex non-linear relationships and interactions among predictors of VE probabilities (11, 12). Although ML approaches cannot provide a well-defined, interpretable result, the SHAP-based explanatory framework was designed to improve the interpretability of the ML models.

In clinical practice, early diagnosis of VE is crucial for improving patient outcomes (5). Although examinations such as lumbar puncture with cerebrospinal fluid analysis and metagenomic next-generation sequencing of cerebrospinal fluid are highly valuable for confirming the diagnosis of encephalitis, these methods have limitations, including invasiveness, high cost, long turnaround times, and limited availability in primary healthcare settings (13, 14). Therefore, identifying high-risk individuals among patients with suspected viral encephalitis in a timely manner to guide further diagnostic testing holds significant clinical value.

The model developed in this study is positioned as a front-end screening tool intended to assist clinicians in decision-making. Based solely on routinely available clinical indicators, including routine blood tests and blood biochemistry, it offers the advantages of low cost and ease of use, making it particularly suitable for emergency departments or resource-limited settings. By performing initial risk stratification, the model can prioritize high-risk patients for early confirmatory testing such as lumbar puncture, thereby optimizing the diagnostic workflow, improving resource utilization, and potentially enhancing patient outcomes.

To address the well-recognized “black-box” challenge associated with machine learning models, we constructed a comprehensive interpretation framework based on SHAP value. SHAP analysis identified albumin and white blood cell counts as the most influential predictors associated with a lower risk of VE. In contrast, neutrophil counts were associated with an increased contribution to VE when neutrophil levels were relatively low. From a clinical perspective, the negative association between albumin levels and the probability of viral encephalitis may reflect hypoalbuminemia as a marker of systemic inflammation, poor nutritional status, or increased vascular permeability—all of which have been linked to adverse outcomes in infectious and neurological diseases (15). Similarly, the observed association between lower white blood cell counts and a higher probability of viral encephalitis is biologically plausible, as viral infections often present with normal or reduced white blood cell counts, whereas bacterial infections typically induce leukocytosis. This pattern has been reported in previous studies examining the laboratory features of viral encephalitis and central nervous system infections (10). Beyond individual predictors, the interaction heatmap revealed a potential synergistic effect between albumin and white blood cell counts, whereby patients with concurrently high levels of both markers consistently fell into the low-risk region for viral encephalitis. Such interaction effects are difficult to detect and quantify using traditional regression methods, but can be naturally captured by tree-based machine learning models. This finding supports the notion that risk assessment for viral encephalitis should not rely on isolated laboratory parameters but rather on integrated patterns across multiple indicators. However, SHAP values only indicate the direction and magnitude of each feature’s contribution to the model’s prediction, but do not imply causal relationships. Thus, we describe these as predictive associations rather than risk or protective factors, which should be interpreted more cautiously in terms of biological interpretation.

## Limitation

Several limitations should be acknowledged. First, the study was conducted at a single center, which may limit the generalizability of the findings. Although cross-validation and test sets were both used, the small sample size of the test set limited the generalizability of our models. External validation using multicenter cohorts is warranted in further study. Second, the proportion of VE cases was relatively high compared with non-VE cases, which may differ from real-world screening populations and could influence predictive values. To address the data imbalances, the ROC-AUC was used to evaluate the performance of our model, as it is robust to such imbalances. Besides, patients without VE were suspected of having VE rather than healthy people, which also led to potential biases for model performance. However, it was applicable in clinical practice, where the main demands was to diagnose the real VE among these potential VE patients. Thus, it should be emphasized that our model was used only for preliminary screening for VE due to the potential biases from differential diagnostic confounding. Third, only 171 patients in the VE group were validated by next-generation sequencing (NGS) testing, and 68 were tested as positive. However, the sensitivity of NGS testing was insufficient, so the diagnoses in our study were based on only International Encephalitis Consortium diagnostic criteria, as clinical practice did. Fourth, although SHAP analysis improves interpretability, it only provides a numerical contribution of predictors on the predicted value rather than establishing causality or biological association. Any interpretability of the SHAP values on biological processes was not suggested. Fifth, due to the retrospective design and too long data collection period, certain clinically relevant variables (e.g., CRP and platelets) could not be systematically obtained and were therefore not included in the model. Moreover, potential time-based biases may also affect the performance of our model. Future prospective studies should incorporate these variables to improve our model.

## Conclusion

In this study, the XGBoost model demonstrated excellent performance in predicting the risk of VE (AUC = 0.949, 95%CI = 0.921 ~ 0.978) in 10-fold cross-validation in the train and test sets (AUC = 0.900, 95% CI = 0.801 ~ 1.000). Based on routine blood tests and blood biochemistry indicators, it can serve as a front-end screening tool suitable for use in emergency departments or resource-limited settings. Combined with the SHAP-based explanatory framework, the analysis identified albumin and white blood cell counts as key protective factors with a synergistic effect. Overall, this model provides an effective and interpretable approach to early risk stratification in patients with suspected viral encephalitis.

## Data Availability

The raw data supporting the conclusions of this article will be made available by the authors, without undue reservation.

## References

[1] Venkatesan A. Encephalitis: intersections between infections and autoimmunity. Clin Microbiol Infect. (2025) 31:529–33. doi: 10.1016/j.cmi.2024.11.028, 39581544

[2] Duerlund LS Nielsen H Bodilsen J. Current epidemiology of infectious encephalitis: a narrative review. Clin Microbiol Infect. (2025) 31:515–21. doi: 10.1016/j.cmi.2024.12.025, 39725074

[3] Matthews R Sargent BF McKeever S Huang Y Ellul MA Michael BD. Viral encephalitis - update on pathogenesis and treatment. Curr Opin Neurol. (2025) 38:388–96. doi: 10.1097/WCO.0000000000001384, 40466008

[4] Gundamraj V Hasbun R. Viral meningitis and encephalitis: an update. Curr Opin Infect Dis. (2023) 36:177–85. doi: 10.1097/QCO.0000000000000922, 37093042

[5] Yong HYF Pastula DM Kapadia RK. Diagnosing viral encephalitis and emerging concepts. Curr Opin Neurol. (2023) 36:175–84. doi: 10.1097/WCO.0000000000001155, 37078655

[6] Haug CJ Drazen JM. Artificial intelligence and machine learning in clinical medicine. Reply N Engl J Med. (2023) 388:2398–9. doi: 10.1056/NEJMra2302038 37342938

[7] Basha S Ks P Chattopadhyay A Pai AR Mahato KK. Artificial intelligence and machine learning in neurodegenerative disease management: a 21st century paradigm. Neurobiol Dis. (2026) 220:107307. doi: 10.1016/j.nbd.2026.107307, 41644015

[8] Xu C Zhao LY Ye CS Xu KC Xu KY. The application of machine learning in clinical microbiology and infectious diseases. Front Cell Infect Microbiol. (2025) 15:1545646. doi: 10.3389/fcimb.2025.1545646, 40375898 PMC12078339

[9] Cheah BCJ Vicente CR Chan KR. Machine learning and artificial intelligence for infectious disease surveillance, diagnosis, and prognosis. Viruses. (2025) 17:882. doi: 10.3390/v17070882, 40733500 PMC12299889

[10] He Q Wang S Chen H Long L Xiao B Hu K. The neutrophil-to-lymphocyte and monocyte-to-lymphocyte ratios are independently associated with clinical outcomes of viral encephalitis. Front Neurol. (2022) 13:1051865. doi: 10.3389/fneur.2022.1051865 36712460 PMC9874857

[11] Al Meslamani AZ Sobrino I de la Fuente J. Machine learning in infectious diseases: potential applications and limitations. Ann Med. (2024) 56:2362869. doi: 10.1080/07853890.2024.2362869, 38853633 PMC11168216

[12] Wei L Wu B Guo T Ru D Gao C Wu J . Development and validation of a machine learning-based model for 90-day prognosis outcome in spontaneous intracerebral hemorrhage patients based on non-contrast computed tomography: a multicenter retrospective observational study. EClinicalMedicine. (2025) 88:103507. doi: 10.1016/j.eclinm.2025.103507, 41181845 PMC12572785

[13] Buddle S Torres Montaguth OE Morfopoulou S Breuer J Brown JR. The use of metagenomics to enhance diagnosis of encephalitis. Expert Rev Mol Diagn. (2025) 25:245–62. doi: 10.1080/14737159.2025.2500655, 40329854

[14] Olie SE Staal SL van de Beek D Brouwer MC. Diagnosing infectious encephalitis: a narrative review. Clin Microbiol Infect. (2025) 31:522–8. doi: 10.1016/j.cmi.2024.11.026, 39581538

[15] Wiedermann CJ. Hypoalbuminemia as surrogate and culprit of infections. Int J Mol Sci. (2021) 22:4496. doi: 10.3390/ijms22094496, 33925831 PMC8123513

