# Supplementary material for: Explainable machine learning-based preliminary screening for viral encephalitis by blood routine analysis
Source: Front Neurol. 2026 Jun 19;17:1844506. doi: 10.3389/fneur.2026.1844506 (PMC13332487; doi:10.3389/fneur.2026.1844506)
Supplement: Supplementary file 1 [file Supplementary_file_1.docx]

## Explainable Machine Learning based Preliminary Screening for Viral Encephalitis by Blood Routine Analysis

Bo Lv^1^, Jie Pan^1*^, Aiming Shi^1*^, Dongxing Wang^2*^

1. Department of Pharmacy, The Second Affiliated Hospital of Soochow University, Suzhou 215004, China

2. ‌Department of Neurology‌, The Second Affiliated Hospital of Soochow University, Suzhou 215004, China

*Corresponding author:

Jie Pan ([pankypan@163.com](mailto:pankypan@163.com))

Aiming Shi (sam740411@163.com);

Dongxing Wang ([wangdongzing073518@163.com](mailto:wangdongzing073518@163.com))

## Supplementary Materials


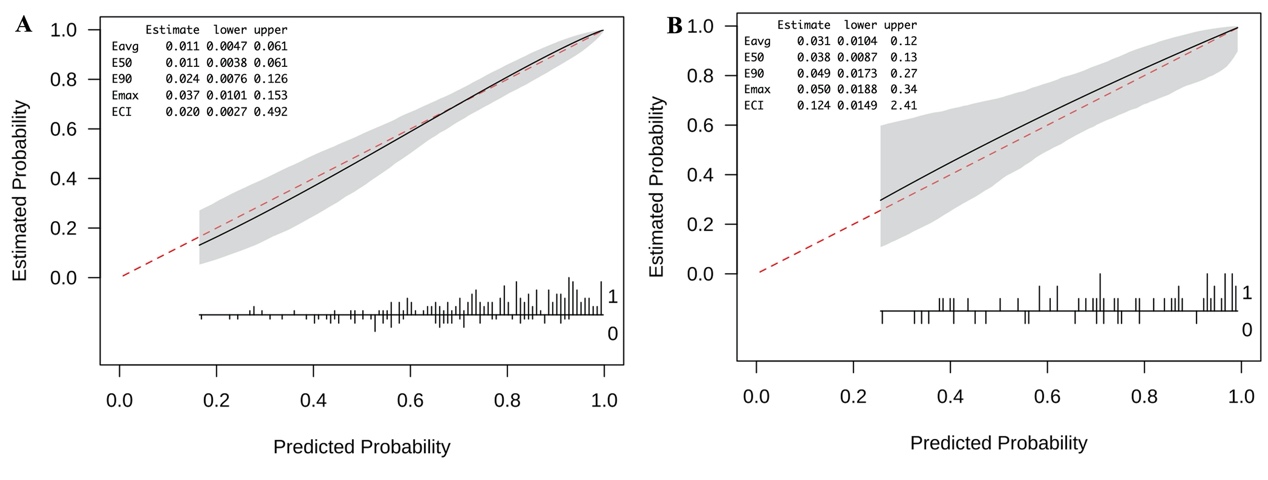


Fig S1. Calibration metric of Logistic regression with 95% confidence interval in

train (A) and test set (B)


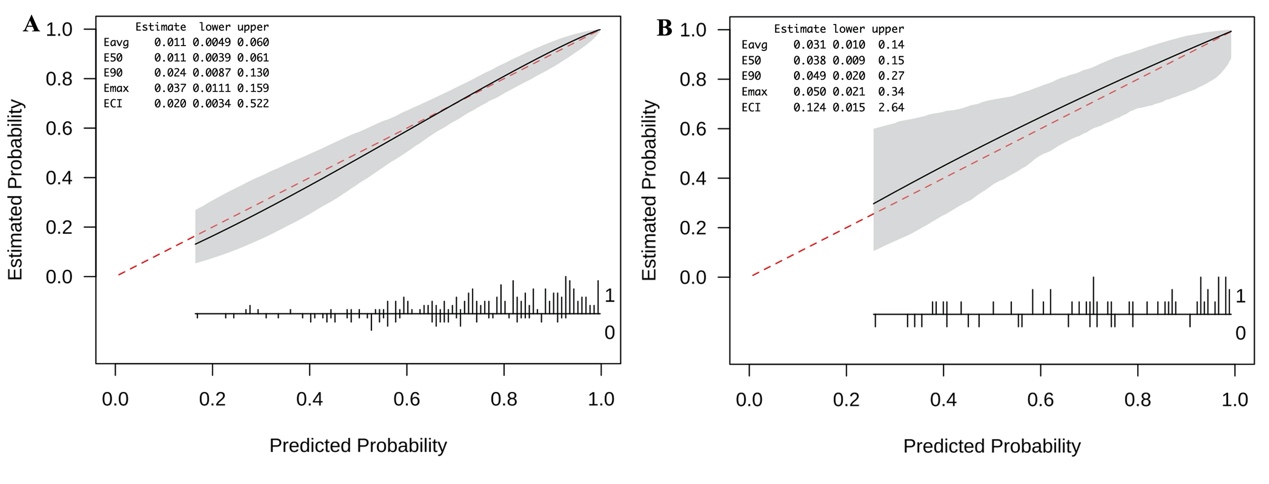


Fig S2. Calibration metric of Knn regression with 95% confidence interval in

Train (A) and test (B) set


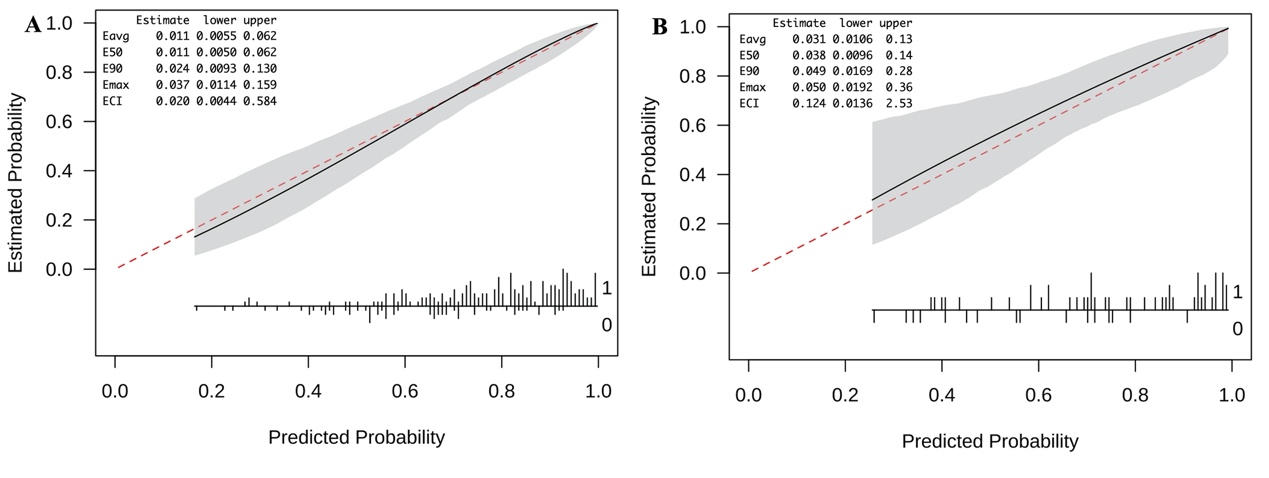


Fig S3. Calibration metric of Random forest regression with 95% confidence interval in

train (A) and test (B) set


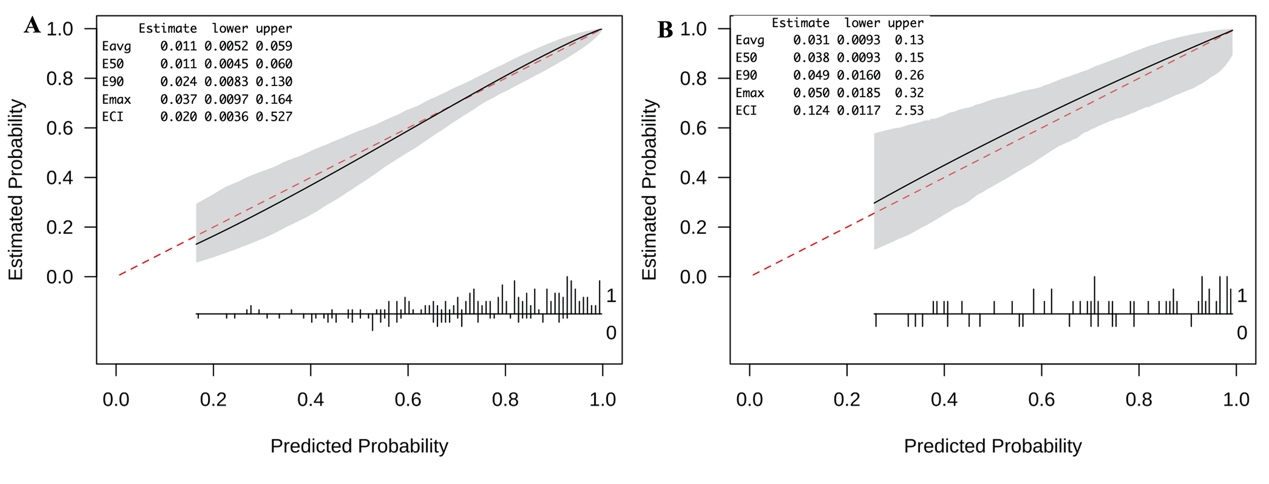


Fig S4. Calibration metric of Svm regression with 95% confidence interval in

train (A) and test (B) set


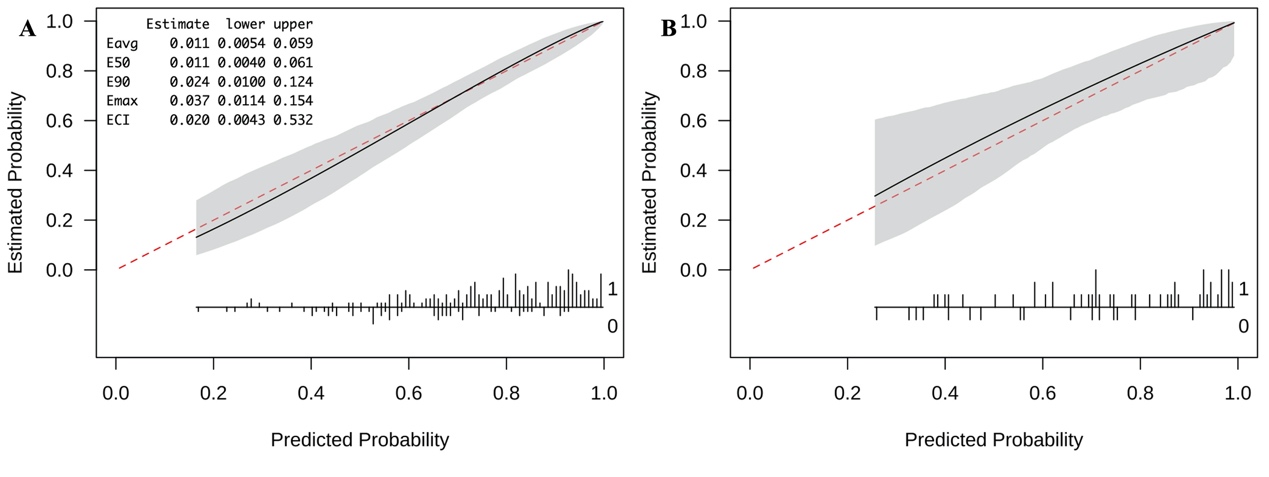


Fig S5. Calibration metric of Xgboost regression with 95% confidence interval in

train (A) and test (B) set


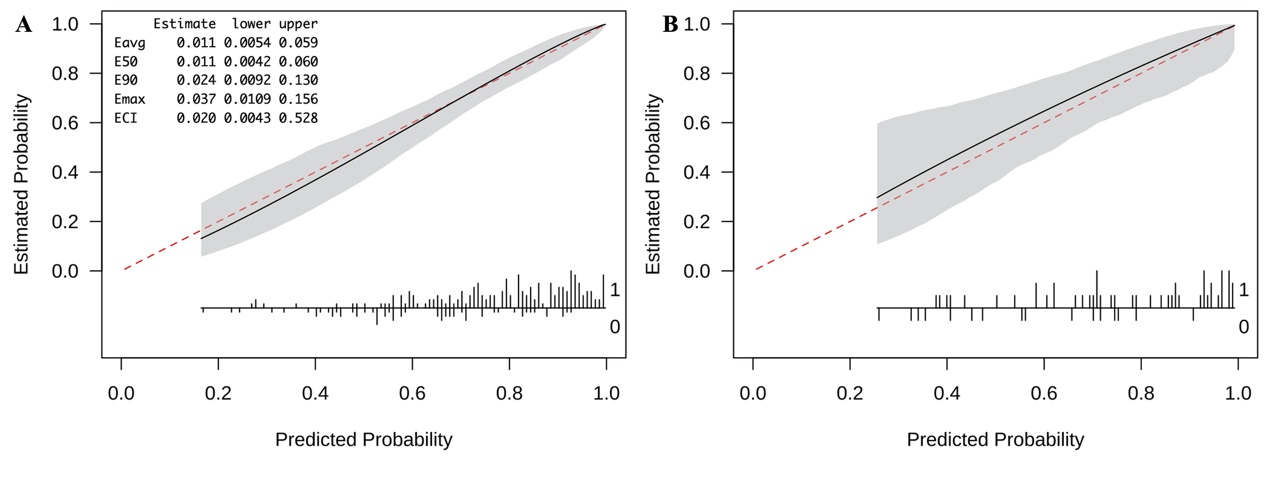


Fig S6. Calibration metric of Xgboost regression with 95% confidence interval in

train (A) and test (B) set
